# Extrinsic- and intrinsic-dependent variation in component communities and patterns of aggregations in helminth parasites of great cormorant (*Phalacrocorax carbo*) from N.E. Poland

**DOI:** 10.1007/s00436-013-3714-7

**Published:** 2013-12-03

**Authors:** Gerard Kanarek, Grzegorz Zaleśny

**Affiliations:** 1 Ornithological Station, Museum and Institute of Zoology Polish Academy of Sciences, ul. Nadwiślańska 108, 80-680 Gdańsk, Poland; 2 Institute of Biology, Department of Invertebrate Systematics and Ecology, Wrocław University of Environmental and Life Sciences, ul. Kożuchowska 5b, 51-631 Wrocław, Poland

## Abstract

In the present study, we investigate the effect extrinsic (habitat and season) and intrinsic (host’s age and sex) factors on the richness, diversity, and structure of parasite component communities and aggregation patterns in the helminth fauna of the great cormorant *Phalacrocorax carbo* from northeastern Poland. The helminth fauna of cormorants from the brackish water habitat was far richer (30 species) than in those from freshwater lakes (18 species) and strongly depend on season and age of the host. The values of diversity index strongly varied in relation to habitat and host age with clear seasonal differences in the value of diversity index, i.e., its value increased over time in adults from the brackish water habitat and decreased in those from the freshwater lakes. The number of helminths in adult and immature birds varied, depending on the season and habitat: in the brackish water habitat, the overall percentage of helminths was higher in spring than in summer, while in the freshwater habitat a higher proportion of helminths was recorded in summer. During spring, in the brackish water habitat, we observed a higher level of aggregation (for all groups of helminths) than in autumn. The opposite pattern was found in the freshwater habitat. However, this regularity was typical of adult birds only. In immature hosts, the level of aggregation was not predictable and varied among the higher taxa. Our study clearly showed that processes determinate diversity, structure, richness, and patterns of aggregation in helminth assemblages of avian hosts are multi-origin and highly complex.

**Electronic supplementary material:**

The online version of this article (doi:10.1007/s00436-013-3714-7) contains supplementary material, which is available to authorized users.

## Introduction

Parasitic assemblages are highly structured groups, composed of many species and shaped by an array of ecological and evolutionary factors. Because of their complexity, precise identification of processes, which determine the structure and diversity of the helminth communities, as well as distribution of parasites among host populations are still among the crucial problems in parasite ecology. In the past decade, a number of papers described the effects of several extrinsic (geographical distribution of host species, quality and diversity of habitat, season, etc.) and intrinsic (host’s age and sex, migrations, etc.) factors on the formation and functioning of helminth assemblages. They primarily focused on the analysis of temporal and spatial variability of helminth communities of easily available hosts: fishes (e.g., Seifertová et al. 2008; Timi et al. 2010; Pérez-del-Olmo et al. 2011) and small mammals (e.g., Goüy de Bellocq et al. 2003; Behnke et al. 2008a, b).

To date, relatively little attention was paid to comprehensive analyses of the processes governing the structure, richness, and diversity of internal parasite assemblages in avian hosts. The helminth communities of birds, especially those closely associated with water, are generally far richer and more diverse than those of fish and mammal hosts (Kennedy et al. 1986; Bush et al. 1990; Poulin 1997), and the processes which determine their composition are definitely more complicated (e.g., Bykhovskaya-Pavlovskaya 1962; Dogiel 1962; Bush 1990). Most of the studies dealing with the variation of structure, richness, and diversity of helminth assemblages in avian hosts overlooked key interactions between the various levels of organization of parasitic assemblages, especially between the component- and infracommunities. Each of these levels developed under different evolutionary and ecological pressures, and only their parallel analysis allows a precise identification of factors determining the structure and diversity of parasite communities. Component communities (assemblages of populations of all parasite species exploiting some subset of host individuals—Bush et al. 1997) are relatively long-lived (their lifespan is equal to the duration of the host population) and therefore relatively stable; they are formed over evolutionary time scales as a result of speciation, extinction, colonization, and host switching (Poulin 2007a). Infracommunities (all parasites of different species in the same host individual—Bush et al. 1997) are relatively short-lived (duration limited to the host individual’s lifespan) and show the greatest variation. They are mainly affected by ecological factors and result from acquisition of new parasite individuals and from their demographic consequences (Poulin 2007a). However, attempts at a holistic approach to the analysis of processes shaping the diversity and structure of the helminth assemblages in avian hosts, taking into account all helminth taxa, hierarchical structure of parasite assemblages and full range of variability, arising from biology and ecology of the birds, are few. Previous studies were based on small samples of birds (Edwards and Bush 1989; Bush 1990) or on host species (e.g., sedentary species with less varied diet and poor helminth fauna—Calvete et al. 2003, 2004) whose characteristics did not reflect the full complexity of the processes forming their helminth assemblages.

Detailed knowledge of factors determining the aggregated distribution of helminths among their host populations is crucial to understanding the processes shaping the structure and functioning of parasite communities. Some authors proposed that aggregated distribution of parasites should be regarded as a characteristic feature of parasitism, widely observed among several groups of macroparasites (Crofton 1971; Shaw and Dobson 1995; Poulin 2007b). Generally, the aggregated distribution of parasites may be caused by heterogeneity of the hosts (Wilson et al. 2002; Poulin 2007a; Matthee and Krasnov 2009) or by demographic processes in the parasite populations (Anderson and Gordon 1982; Poulin 2007b). Host-dependent factors have been suggested as more important in generating aggregative patterns than are parasite-related mechanisms (Wilson et al. 2002; Matthee and Krasnov 2009), but this subject is still debated (Morand and Krasnov 2008). When aggregation patterns are similar between different helminth species exploiting the same host species and host population, these patterns are governed by the host’s biology. When, however, these patterns are produced by parasite-related differences, they should vary among parasite species (Matthee and Krasnov 2009; Marques et al. 2010; Pérez-del-Olmo et al. 2011), showing differences in their life strategies; for example, food-transmitted parasites exhibit a more pronounced aggregation (Bush et al. 1993; Marcogliese 2007). Furthermore, when the main patterns are similar in different taxa, but are pronounced to different degrees, then the differences are likely to be produced by among-parasite differences in life history, but the causes underlying such differences are not well understood (Morand and Krasnov 2008). This clearly shows that detailed studies comprising both aggregation patterns among different parasite taxa infecting a single host population at a given time and place, and different host populations and/or groups of hosts with different characteristics (age, sex, breeding status, etc.) are necessary to understand these processes. In recent literature, such analyses concerning helminth parasites are extremely rare (Newey et al. 2005; Marques et al. 2010); in avian hosts, the issue has been investigated only to a limited extent (Kanarek 2011; D’Ávila et al. 2012).

In this paper, we investigate the effect of several extrinsic (habitat and season) and intrinsic (host’s age and sex) factors on the richness, diversity, and structure of parasite component communities and aggregation patterns in the helminth fauna of the great cormorant *Phalacrocorax carbo* from northeastern Poland. To our knowledge, the presented work is one of the few investigations based on a large sample of birds and considering the full range of variation resulting from the host’s biology and ecology and the hierarchical structure of parasite assemblages.

## Materials and methods

### Study area, sampling, and processing protocols

In 2001–2005, a total of 491 great cormorants *P. carbo sinensis* (Blumenbach, 1798) of different ages (209 nestlings, 202 adults, and 80 immature individuals) from northeastern Poland were subject to full helminthological examination. Two locations (breeding colonies on Lake Wulpińskie 53°21′N, 19°14′E and Lake Selment Wielki 53°47′N, 22°31′E) represented typical inland freshwater habitats; another two were typically estuarine and brackish (breeding colony at Kąty Rybackie on the Vistula Spit 54°21′N, 19°14′E and the Vistula Lagoon 54°35′N, 19°48′E). The study areas were described in detail by Kanarek (2009). Adult and immature birds were shot before the breeding season (March–April) and in summer (July–August), after the end of the breeding season. Nestlings were collected from nests located in selected breeding colonies (May–June). Based on the plumage, the birds were classified as either adult (sexually mature) or immature individuals (individuals which had left the nest, but had not reached sexual maturity). Sex was determined by gonad inspection only in the adult and immature individuals; the degree of gonad development precluded sexing of the chicks. The structure of the sample in terms of habitat, age, sex, and season is presented in Table 1.

**Table 1:** Number of *Ph. carbo* specimens examined by habitat, age, season, and sex

Habitat	Season	Adult			Immature			Chicks
		Female	Male	Total	Female	Male	Total	
Brackish water	Spring	34	42	76	11	12	23	
	Summer	16	16	32	13	2	15	
	Combined	50	58	108	24	14	38	144
Freshwater	Spring	14	35	49	1	0	1	
	Summer	9	36	45	19	22	41	
	Combined	23	71	94	20	22	42	65
Total		73	129	202	44	36	80	209

The birds were then transported to the laboratory and immediately examined for parasites or frozen (−20 °C) for later necropsy. Full-body necropsy followed the commonly accepted protocols. Prior to identification, the helminths were washed in physiological salt solution, counted, fixed and preserved in 70 % ethanol, and then processed according to the standard techniques; digeneans, tapeworms, and acanthocephalans were stained with alcohol borax carmine, dehydrated, cleared, and mounted in Canada balsam. Nematodes were cleared in glycerine or lactophenol and, after identification, transferred to 70 % ethanol. Voucher specimens are deposited in the Polish Collection of Parasitic Helminths, Museum of Natural History, Wrocław University, Poland.

### Ecological terminology and indices of community structure

The ecological terms used here follow Bush et al. (1997). The helminth species were divided into three ecological groups: cormorant specialists, generalists, and captured specialists. The cormorant specialists were defined as species which mature mainly in cormorants or in other members of the family Phalacrocoracidae, the generalists as species which grow to adulthood in a wide variety of avian species of different families and in fish-eating mammals, and the captured specialists as those with narrow host specificity, for example birds of families other than Phalacrocoracidae or marine mammals. Classification of helminth species to a specific ecological group is often problematic and depends mainly on the local compound community structure (Edwards and Bush 1989; Bush 1990). For this classification, we used data obtained during a comprehensive analysis of helminth communities of several species of water and wading birds from northern Poland (Kanarek, unpublished data), and the data from Central Europe contained in the studies of Sitko et al. (2006), Sitko and Okulewicz (2010), and Sitko (2011).

In order to measure the indices of component community richness and diversity, observed helminth species richness, estimated species richness, Simpson’s Index of Dominance, and Brillouin’s Index of Diversity were calculated. Because the observed species richness of component communities depends on the sampling effort and the presence of rare species in the analyzed sample, species richness should be estimated (see review in Poulin 2007a). To assess the estimated species richness of the component communities, we calculated three non-parametric estimators, recommended for analysis of parasite communities: Chao1, first-order Jacknife estimator (Jack1), and bootstrap estimator (Boot) (e.g., Poulin 1998; de la Luz Romero-Tejeda et al. 2008; for detailed description of these estimators see Colwell 2009). Two hundred randomizations with replacement were generated for each component community and for pooled data; the software used was EstimateS v. 8.2 (Colwell 2009). Statistical significance of differences between the observed and estimated species richness was tested with chi-square test. Simpson’s Index of Dominance and Brillouin’s Index of Diversity were calculated for each component community and for pooled data. Simpson’s Index of Dominance (Magurran 2004) was calculated as *D* = Σ[*n*_*i*_(*n*_*i*_ − 1)/*N*(*N* − 1)], where *n*_*i*_ is the number of individuals of species *i* and *N* is total number of helminths in the community. Brillouin’s Index of Diversity (Legendre and Legendre 1998) was calculated using Stirling’s approximation (Zar 1996) as *H* = 1/*N* log (*N*!/*n*_1_!*n*_2_!*n*_3_!…*n*_*s*_!), where *N* is the total number of helminths and *n*_1_, *n*_2_, *n*_3_… *n*_*s*_ are the numbers of specimens of 1, 2, 3… *i* species in the analyzed community.

For qualitative comparisons between the analyzed communities, Jaccard’s Index of Similarity was used [*J* = *c*/(*a* + *b* + *c*), where *a* is the number of parasite species in the first community, *b* is the number of parasite species in the second community, and *c* is the number of helminth species common to both communities]. For quantitative comparisons, we used Steinhaus’s Index, calculated as *S* = *2W*/(*A* + *B*) where *A* and *B* are the sums of abundances of all helminths in the compared communities, and *W* is the sum of the minimum abundances of the various species, this minimum being defined as the abundance in the community where the species is the rarest. Both indices were used as described by Legendre and Legendre (1998).

### Statistical analysis and aggregation parameters

The percentage distribution of higher taxa of helminths (expressed as presence/absence) was also analyzed with maximum likelihood techniques based on log-linear analysis of contingency tables using Statistica 9.1 software. The analysis was initiated with the most complex model involving all possible effects (site, season, and host sex) and interactions. Because of the lack of seasonal variation and the impossibility to sex the nestlings, they were excluded from this analysis. Next, the minimum sufficient model was generated where chi-square result was not significant, indicating that the model was adequate in explaining the data. Log-linear analysis for the presence of nematodes was performed with exclusion of *Contracaecum rudolphii* due to its 100 % prevalence within all adult and immature birds (for details see Kanarek 2011).

To characterize the aggregation patterns in the most abundant higher taxa of helminths (Digenea, Cestoda, and Nematoda—Acanthocephala were excluded from the analysis because of their low abundance) in relation to selected factors, we used variance-to-mean ratio (VMR) and D index of discrepancy as the index of aggregation (for details see Poulin 2007a). The aggregation indices were calculated using the software package Quantitative Parasitology v. 3.0 (Reiczigel and Rózsa 2005). The level of aggregation was also assessed with the parameter *b* of Taylor’s Power Law (Taylor 1961). For that purpose, the log variance of the mean abundance was plotted against the log mean of abundance (both calculated separately for each site and age class) and the parameter *b* was estimated with the regression coefficient of the regression line. Statistical analysis of regression was performed using Statistica v. 9.1 whereas dot graph, with line fitting by least square methods, was made using Microsoft Office Excel 2007.

## Results

In total, 31 helminth species were recorded. Among these, 14 were classified as cormorant specialists (*Hysteromorpha triloba*, *Holostephanus dubinini*, *Paryphostomum radiatum*, *Petasiger exaeretus*, *Petasiger phalacrocoracis*, *Paradilepis scolecina*, *Cyathostoma microspiculum*, *Contracaecum rudolphii*, *Syncuaria squamata*, *Desmidocercella incognita*, *Eustrongylides excisus*, *Baruscapillaria carbonis*, *Baruscapillaria rudolphii*, and *Andracantha phalacrocoracis*), nine as generalists (*Stephanoprora pseudoechinata*, *Cercarioides aharonii*, *Cryptocotyle concava*, *Metagonimus yokogawai*, *Metorchis xanthosomus*, *Diphyllobothrium ditremum*, *Ligula intestinalis*, *Schistocephalus solidus*, and *Southwellina hispida*), and eight as captured specialists. The group of captured specialists included two species of grebe specialists (Podicipedidae—*Echinochasmus coaxatus* and *Echinochasmus spinulosus*), two duck specialists (Anatidae—*Apatemon gracilis* and *Polymorphus minutus*), and two marine mammal specialists (Cetacea, Pinnipedia—*Anisakis simplex* and *Corynosoma semerme*); one was characteristic of herons (Ardeidae—*Posthodiplostomum cuticola*) and one of gulls (Laridae—*Cosmocephalus obvelatus*) (for details see Supplementary material).

In total, 381,035 helminth individuals were obtained, including 225,949 Cestoda, 101,544 Digenea, 53,468 Nematoda, and 74 Acanthocephala. Most of the collected helminth species were detected in the intestine, except five species found in the proventriculus and gizzard (*A. simplex*, *C. rudolphii*, *C. obvelatus*, *S. squamata*, and *E. excisus*), one each in the gall bladder (*M. xanthosomus*), trachea (*C. microspiculum*), and air sacks (*D. incognita*).

### Habitat-dependent changes in the helminth community

The quantitative composition of the helminth fauna depended strongly on the habitat: the majority of the helminths (253,925 specimens; 66 % of all helminths) were detected in hosts from the freshwater habitat; a higher proportion of tapeworms was found in birds from lakes Wulpińskie and Selment Wielki, whereas in the Vistula Lagoon and the Vistula Spit the cormorant helminth fauna was dominated by trematodes and nematodes (Table 2).

**Table 2:** Percentage distribution of higher taxa of helminths by habitat and age of the host

	Habitat	Age category			
		Adult	Immature	Chicks	Total
All helminths	Brackish water	41	18.7	39.3	33.4
	Freshwater	59	81.3	60.7	66.6
	Combined	43	32.4	23.7	100
Digenea	Brackish water	84.9	52.9	61.7	70.4
	Freshwater	15.1	47.1	38.3	29.6
	Combined	45	19.8	35	100
Cestoda	Brackish water	15.5	8.9	0.03	10.3
	Freshwater	84.5	91.1	99.9	89.7
	Combined	42.6	41.2	16.2	100
Nematoda	Brackish water	60.2	42.1	73.5	62.1
	Freshwater	39.8	57.9	26.5	37.9
	Combined	47.5	18.1	34.4	100
Acanthocephala	Brackish water	54	22.2	5.3	51.3
	Freshwater	46	77.8	–	48.7
	Combined	85.1	12.2	2.7	100

The helminth fauna of cormorants from the brackish water habitat was far richer (30 species) than in those from lakes Selment Wielki and Wulpińskie (17 species) (Table 3). Seventeen species were common to both fresh- and brackish-water component communities. Except for one captured specialist (*P. cuticola*) and two generalists (*M. xanthosomus* and *L. intestinalis*), all of them were cormorant specialists (*H. triloba*, *H. dubinini*, *P. radiatum*, *P. exaeretus*, *P. phalacrocoracis*, *P. scolecina*, *C. microspiculum*, *C. rudolphii*, *S. squamata*, *D. incognita*, *E. excisus*, *B. carbonis*, *B. rudolphii*, and *A. phalacrocoracis*) (for details see Supplementary material). Thirteen species were exclusive to the brackish water component community (*A. gracilis*, *E. coaxatus*, *E. spinulosus*, *S. pseudoechinata*, *C. aharonii*, *C. concavum*, *M. yokogawai*, *D. ditremum*, *S. solidus*, *A. simplex*, *C. obvelatus*, *C. semerme*, and *S. hispida*), while only one was exclusive to the freshwater habitat (*P. minutus*). The numbers of species classified as cormorant specialists were similar in the brackish water (15) and freshwater habitats (14). In contrast, the number of generalists and captured specialists in the brackish water component community (eight species) was much higher than in the freshwater habitat (two species) (for details see Supplementary material). The captured specialists and generalists representing Digenea showed a distinct habitat-dependent variation: the first group was recorded mainly in the freshwater habitat (25.7 % vs. 2.1 %; *χ* ^2^ = 33.865; *P* < 0.001), while the prevalence of generalists was two times higher in the brackish water habitat (52.7 % vs. 28.7 %; *χ* ^2^ = 16.838; *P* <0.001). Furthermore, the prevalence of generalists was significantly higher in spring than in summer (47.6 % vs. 33.8 %; *χ* ^2^ = 5.540; P <0.018). No such differences were observed for the group of cormorant specialists (*χ* ^2^_SITE_ = 1.547, *P* < 0.213; *χ* ^2^_SEASON_ = 1.108, *P* < 0.292). No differences in the level of infection between adult and immature birds were observed for any of the three groups of digeneans. A similar pattern was displayed by the tapeworms: both specialists and generalists showed a habitat-dependent variation with the dominance of specialists in the freshwater sites (100.0 % vs. 93.8 %; *χ* ^2^ = 8.659; *P* < 0.003) and of generalists in the brackish-water habitat (32.9 % vs. 2.9 %; *χ* ^2^ = 41.955; *P* < 0.001). Moreover, we observed a season-biased distribution of the generalist tapeworms whose prevalence was six times higher in spring than in summer (30.2 % vs. 5.3 %; *χ* ^2^ = 29.059; *P* < 0.001). None of the tapeworm species was classified as a captured specialist. The pattern observed for the nematodes was different—they showed no statistically significant dependence on any analyzed factor.

**Table 3:** Measures of component community structure by habitat and host age

	Habitat	Age category			
		Adult	Immature	Chicks	Total
Observed species richness	Brackish water	22	21	21	30
	Freshwater	17	17	11	18
	Combined	22	25	21	31
Estimated species richness Chao1/Jack1/Boot	Brackish water	21.7/22.3/22.1	18.8/21.1/19.8	20.4^a^/21.4/20	28.7^a^/30.5/29.2
	Freshwater	16.6/17.5/17.1	16.1/17.1/16.6	10.9^a^/11.1/11.1	17.6^a^/18.1/17.9
	Combined	21.9/22.1/22.1	22.3./25.2/23.5	19.8/21.3/20.5	29/31.3/29.8
Simpson’s Dominance Index	Brackish water	0.232	0.262	0.245	0.217
	Freshwater	0.699	0.729	0.484	0.655
	Combined	0.385	0.593	0.263	0.400
Brillouin’s Diversity Index	Brackish water	1.683	1.580	1.684	1.818
	Freshwater	0.685	0.635	1.063	0.789
	Combined	1.321	0.904	1.627	1.329

^a^Calculated using the classic formula (for details see Colwell 2009)

### Age-, sex-, and season-dependent variation in the helminth community

Considering the pooled data, the greatest percentage of parasites was found in adults, followed by immature individuals and chicks (Table 3; *χ* ^2^ = 34.046, *P* < 0.001). The number of helminths in adult and immature birds varied, depending on the season and habitat: in the brackish water habitat, the overall percentage of helminths was higher in spring than in summer (70.8 % vs. 29.2 %), while in the freshwater habitat a higher proportion of helminths was recorded in summer (25.7 % vs. 74.3 %). This pattern was exhibited by almost all the groups of helminths (Digenea brackish water 70.8 % vs. 29.2 %, freshwater 36.7 % vs. 63.3 %; Cestoda brackish water 78.3 % vs. 21.7 %, freshwater 22.9 % vs. 77.1 %; Nematoda brackish water 59.8 % vs. 40.2 %, freshwater 44.1 % vs. 55.9 %); only Acanthocephala showed the opposite tendency (brackish water 91.6 % vs. 8.4 %, freshwater 97.2 % vs. 2.8 %). The overall percentage of helminths found in adult and immature birds was slightly higher in males than in females (61.3 % vs. 38.7 %) for all the groups of helminths (Digenea—56.4 % vs. 43.6 %; Cestoda—62.8 % vs. 37.2 %; Nematoda—62.1 % vs. 37.9 %), except Acanthocephala (44.4 % vs. 55.6 %).

The comparison of the estimated and observed species richness for each combination (see Tables 3, 4, and 5) revealed no statistically significant differences between those values, suggesting that the sample size was sufficient to detect the vast majority of helminth species. The species richness of parasite communities of birds from the brackish water habitat was similar among all three age classes and was much higher than in birds from the freshwater habitat, where the component communities of adult and immature cormorants displayed the same species richness while the lowest number of helminth species was recorded in chicks (Table 3). Nine species of helminths occurred in both types of habitats and in birds of all the age classes. Except for one generalist (*M. xanthosomus*), they were cormorant specialists (*H. triloba*, *H. dubinini*, *P. radiatum*, *P. exaeretus*, *P. phalacrocoracis*, *P. scolecina*, *C. rudolphii*, and *S. squamata*). One species was exclusive to adults (captured specialist—*A. simplex*), three were exclusive to immature birds (two generalists—*C. aharonii* and *M. yokogawai*, and one captured specialist—*P. minutus*), and five were exclusive to chicks (all captured specialists—*A. gracilis*, *E. coaxatus*, *E. spinulosus*, *C. obvelatus*, and *C. semerme*). Five species of parasites, common to adult and immature birds (*E. excisus*, *B. carbonis*, *B. rudolphii*, *A. phalacrocoracis*, and *S. hispida*), were cormorant specialists; one was classified as a generalist (*S. hispida*). Five species were detected in adult and immature cormorants exclusively in spring (*M. yokogawai*, *A. simplex*, *E. excisus*, *A. phalacrocoracis*, and *S. hispida*), but only two in summer (*C. aharonii* and *P. minutus*) (for details see Supplementary material). The largest number of cormorant specialists (14 and 15 species, respectively) and the smallest (two species) number of captured specialists occurred in adult and immature birds. In contrast, the greatest number (six species) of generalists and captured specialists, and the smallest number of cormorant specialists (10) were recorded for chicks (for details see Supplementary material).

**Table 4:** Measures of adult and immature *Ph. carbo* component community structure in relation to habitat and season

	Habitat	Adult		Immature		Combined	
		Spring	Summer	Spring	Summer	Spring	Summer
Observed species richness	Brackish water	18	15	18	13	20	16
	Freshwater	15	13	10^b^	14	15	15
Estimated species richness Chao1/Jack1/Boot	Brackish water	21.6/22.5/22.1	16.9/18.5/17.8	17.9^a^/19.7/18.6	13.2/14.1/13.7	23.2/24.6/23.9	17.6/19.4/18.6
	Freshwater	16.6/17.4/17.1	13.4/14.5/14	–^b^	15.9/17.1/16.5	16.7^a^/17.6/17.2	17.1^a^/18.1/17.4
Simpson’s Dominance Index	Brackish water	0.267	0.198	0.297	0.198	0.264	0.202
	Freshwater	0.581	0.832	0.606	0.735	0.581	0.766
Brillouin’s Diversity Index	Brackish water	1.555	1.756	1.488	1.596	1.563	1.75
	Freshwater	0.909	0.404	0.844	0.612	0.917	0.556

^a^Calculated using the classic formula (for details see Colwell 2009)

^b^Single specimens of host were investigated

**Table 5:** Measures of adult and immature *Ph. carbo* component community structure in relation to season and sex of the host

	Season	Adult			Immature			Total		
		Female	Male	Total	Female	Male	Total	Female	Male	Total
Observed species richness	Spring	21	22	22	17	18	21	21	24	24
	Summer	17	18	20	15	17	18	19	20	23
	Combined	22	22	22	18	23	25	23	25	26
Estimated species richness Chao1/Jack1/Boot	Spring	20.6/22.2/21.5	21.6/22.6/22.1	21.6/22.1/21.9	16.3^a^/17.2/16.3	16.5/18.5/17.5	19.1^a^/21/19.6	20.6/21.7/21.3	23.2^a^/24.5/23.8	23.2^a^/24.2/23.7
	Summer	16.7^a^/17.7/17	16.4^a^/18.5/17.1	18.7/20.4/19.5	14.6/15.3/14.9	15.8/17.3/16.5	16.8/18.2/17.4	18/19.5/18.7	18.8^a^/20.4/19.3	22^a^/23.5/22.3
	Combined	21.3/22.5/22.1	21.7/22.5/22.1	22/22.2/22.1	17.4^a^/18.3/17.8	20.8/23/21.8	22.4/25.5/23.6	22.2/23.5/22.9	23.8/25.1/24.4	24.7/26.1/25.3
Simpson’s Dominance Index	Spring	0.274	0.375	0.321	0.389	0.293	0.314	0.283	0.353	0.319
	Summer	0.435	0.541	0.517	0.678	0.646	0.666	0.617	0.594	0.603
	Combined	0.292	0.443	0.385	0.626	0.563	0.593	0.437	0.481	0.463
Brillouin’s Diversity Index	Spring	1.531	1.304	1.419	1.337	1.488	1.470	1.520	1.354	1.434
	Summer	1.175	1.038	1.077	0.722	0.779	0.752	0.848	0.934	0.905
	Combined	1.489	1.209	1.321	0.843	0.955	0.904	1.20	1.135	1.166

^a^Calculated using the classic formula (for details see Colwell 2009)

The dependence between the species richness and season was very clear in adults: soon after their arrival from the wintering grounds, the species richness was the highest and decreased over time both in the birds from the freshwater habitat, and from the Vistula Lagoon (Table 4). The species richness recorded for males was slightly higher than in females (Table 5).

### Diversity, dominance, and similarity indices

The helminth fauna of birds from the brackish water habitat was more diverse and less dominated compared to birds from the freshwater habitat (Table 3). The highest value of Brillouin’s Index of Diversity was recorded for adults from the Vistula Lagoon, but combining hosts of both types of habitat, the highest diversity and the lowest dominance were recorded in chicks (Table 3). There were no fundamental differences in the diversity or dominance indices between males and females, neither among adult nor among immature hosts (Table 5). We found clear seasonal differences in the value of diversity/dominance indices: the helminth fauna of both adult and immature hosts was more diverse in spring than in summer (Table 5). As in the case of species richness, the value of diversity index increased over time in adults from the brackish water habitat and decreased in those from the freshwater lakes (Table 4). Table 6 shows values of Jaccard’s and Steinhaus’s indices in relation to habitat and host’s age. Jaccard’s index, based on presence/absence data, varied less than Steinhaus’s index which considers abundance of species. Qualitatively (Jaccard’s index), adult and immature hosts were the most similar, chicks and adult/immature birds—the least similar. Quantitatively (Steinhaus’s index), the most similar age classes were adults and chicks, and the least similar age classes were immature birds and chicks (Table 6). The smallest quantitative similarity was recorded for the component communities of chicks from the freshwater habitat and those of adult/immature hosts from the Vistula Lagoon (Table 6). The similarity between adult and immature birds from the fresh- and brackish-water habitats was higher in spring than in summer, both in qualitative (spring brackish water vs. spring freshwater 0.293, spring brackish water vs. summer freshwater 0.288, summer brackish water vs. spring freshwater 0.280, and summer brackish water vs. summer freshwater 0.274) and quantitative (spring brackish water vs. spring freshwater 0.553, spring brackish water vs. summer freshwater 0.345, summer brackish water vs. spring freshwater 0.443, and summer brackish water vs. summer freshwater 0.248) terms.

**Table 6:** Values of Jaccard’s index for qualitative similarity and Steinhaus’s index for quantitative similarity between selected component communities in relation to habitat and age of the hosts

	Jaccard’s index for qualitative similarity						Steinhaus’s index for quantitative similarity					
	Ad F	Imm F	Chic F	F Com	Imm C	Chic C	Ad F	Imm F	Chic F	F Com	Imm C	Chic C
Ad B	0.772	0.695	0.500				0.376	0.351	0.535			
Imm B	0.583	0.520	0.454				0.288	0.346	0.533			
Chic B	0.480	0.461	0.550				0.191	0.209	0.394			
B Com				0.580						0.383		
Ad C					0.807	0.642					0.897	0.620
Imm C						0.533						0.579

Table headers as coded as follows: *Ad B* adult brackish water, *Ad F* adult freshwater, *Imm B* immature brackish water, *Imm F* immature freshwater, *Chic B* chicks brackish water, *Chic F* chicks freshwater, *B Com* brackish water combined, *F Com* freshwater combined, *Ad C* adult combined, *Imm C* immature combined, *Chic C* chicks combined

### Distribution and aggregation parameters

We investigated the presence of higher taxa of helminths (Digenea, Cestoda, Nematoda, and Acanthocephala) in relation to extrinsic and intrinsic factors (habitat, season, age, and sex). The log-linear analyses produced the following models: for Digenea, sex/site × sex/age × season/age/site × Digenea/season/site (*χ* ^2^ = 12.987; *P* = 0.737); for Cestoda, sex/site × sex/age × season/age/site × Cestoda (*χ* ^2^ = 18.980; *P* = 0.523); for Nematoda, sex/age × season/age/site × sex/site × Nematoda (*χ* ^2^ = 23.571; *P* = 0.262); and for Acanthocephala, sex/site × season/age/site × sex/age × Acanthocephala (*χ* ^2^ = 15.567; *P* = 0.743). The results clearly indicate a site- and season-dependent relationship (i.e., adults were more infected during spring, immature hosts in autumn) only for digeneans. The presence of both tapeworms and acanthocephalans at the level of component communities seems not to be affected by the analyzed factors.

**Fig. 1:**
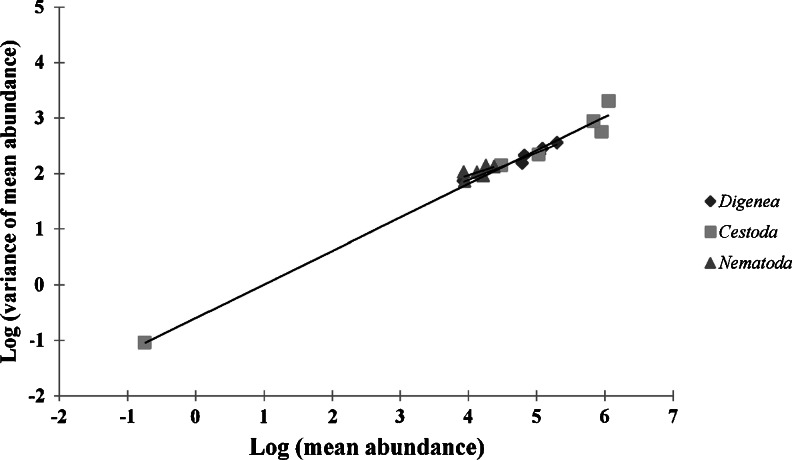
Aggregation level of higher taxa of helminths of *P. carbo* expressed as Taylor’s power relationship

The examination of the relationship between the log variance of mean abundance and the mean abundance of Digenea, Cestoda, and Nematoda in terms of Taylor’s Power Law showed that all the analyzed taxa tended to have aggregated distribution (*b* slope higher than 1). Digeneans were characterized by the highest level of aggregation (*b* = 1.951; *P* < 0.001); the *b* slope for tapeworms was 1.640 (*P* < 0.001), whereas for nematodes the value was relatively low (1.165) indicating a random distribution (Fig. 1). However, ANOVA test (*P* = 0.135) for regression analysis for nematodes was not statistically significant, and two other indices (VMR and D) clearly indicate that nematodes are also characterized by aggregated distribution. Surprisingly, values of both VMR and D closely depended on the habitat, season, age, and sex of the host (Table 7). During spring, in the brackish water habitat, we observed a higher level of aggregation (for all groups of helminths) than in autumn. The opposite pattern was found in the freshwater habitat where the level of aggregation was higher during autumn. However, this regularity was typical of adult birds only. In immature hosts, the level of aggregation was not predictable and varied among the higher taxa (Table 7).

**Table 7:** Values of variance-to-mean ratio (VMR) and index of discrepancy (D) of higher taxa of helminths in relation to habitat (B—brackish water, F—freshwater), season, age, and sex of the host

Aggregation parameter	Taxon	Habitat	Pull.	Immature				Adult			
				Male		Female		Male		Female	
				Spring	Summer	Spring	Summer	Spring	Summer	Spring	Summer
D	Digenea	B	0.694	0.460	–	0.434	0.539	0.457	0.385	0.566	0.381
		F	0.549	–	0.571	–	0.729	0.455	0.691	0.472	0.607
	Cestoda	B	0.952	0.521	–	0.602	0.575	0.532	0.470	0.508	0.491
		F	0.690	–	0.273	–	0.286	0.450	0.478	0.393	0.436
	Nematoda	B	0.674	0.269	–	0.251	0.383	0.511	0.343	0.336	0.351
		F	0.612	–	0.498	–	0.495	0.426	0.568	0.318	0.338
VMR	Digenea	B	401.91	603.77	–	206.74	419.12	236.38	360.40	930.71	107.62
		F	318.15	–	402.79	–	732.20	85.87	103.54	114.44	45.39
	Cestoda	B	2.00	390.67	–	661.80	172.38	289.45	145.82	138.06	106.79
		F	1597.77	–	506.44	–	644.69	591.43	1,025.37	397.45	775.96
	Nematoda	B	181.31	27.67	–	22.70	124.17	216.16	99.79	46.24	58.69
		F	121.29	–	193.96	–	153.28	127.83	151.74	23.44	77.04

## Discussion

The helminth fauna of *Ph. carbo* from northeastern Poland is rich in species and individuals which is characteristic of parasite assemblages of most water and wetland birds (Bush and Holmes 1986; Stock and Holmes 1987; Edwards and Bush 1989). The overall value of Brillouin’s Diversity Index (1.329) is higher than in other bird species and approaches those observed in mammals (Kennedy et al. 1986; Kennedy and Bakke 1989). The variability of structure, richness, and diversity of helminth component communities in avian hosts in relation to some extrinsic and intrinsic factors has been poorly documented; however it is not surprising that extrinsic rather than intrinsic factors play a major role in structuring and functioning of helminth assemblages (Behnke et al. 2008a, b). The results presented in this paper clearly show that the variation in richness, diversity, and structure of component communities of the cormorant helminth assemblage are governed by both extrinsic and intrinsic factors, with the major role played by habitat, season, and host’s age, whereas host’s sex is of only minor significance. Moreover, habitat, season, and host’s age are not interdependent but interact strongly. The results are only partly compatible with other investigations which point exclusively to habitat as the most important factor affecting the structure, richness, and diversity of helminth component communities of avian hosts; other factors are irrelevant (Edwards and Bush 1989; Bush 1990; Calvete et al. 2003, 2004; Violante-González et al. 2011). Several authors have reported the effect of host’s age on the occurrence of helminths in birds (Bakke 1972; Moore et al. 1987; Sitko 1993; Isomursu et al. 2006), as well as on seasonal changes related to migrations (Tallman et al. 1985; Wallace and Pence 1986; Ewart and McLaughlin 1990), but host’s sex has been found to be relevant only in few cases (Álvarez et al. 2006; Isomursu et al. 2006; Monteiro et al. 2011). Unfortunately, the observation is not supported at the component community level. Likewise, we have found extremely complex and inconsistent relationships between the values of aggregation parameters in the higher taxa of helminths. The distribution of all the analyzed helminth taxa is aggregated, and the values of aggregation indices vary depending on habitat, season, age, and sex of the hosts. Aggregated distribution of parasites among hosts is mainly a consequence of individual variation in susceptibility to helminth invasions, resulting from behavioral, physiological, and immunological differences among the hosts. An important role in the observed distributions may have been played by other factors arising from the seasonal variation in parasite recruitment by birds, perhaps by habitat heterogeneity, or by dissimilarities in distributions of potential intermediate or paratenic hosts (Karvonen et al. 2004; Knudsen et al. 2004). A thorough explanation of these relationships at the level of higher helminth taxa requires a more detailed analysis at the level of particular species. The highest values of aggregation parameters have been recorded in chicks, relative to immature and adult birds. This relationship may be a consequence of much greater homogeneity in the population of adult and immature birds, resulting from their longer life expectancies: individuals most susceptible to parasitic infection may have been eliminated from the population. This may suggest that chicks are the most heterogeneous group, with high proportion of uninfected birds and birds with low mean infection intensities in the early days of life, and more heavily infected individuals just before fledging (Kanarek 2011). The host-dependent origin of aggregation variation is also suggested by the relationship between the values of aggregation and host’s sex, but the trend of this variation and its strength are not unambiguous. Male and female cormorants usually vary only slightly in their feeding ecology: males, being heavier, dive deeper (Kato et al. 1999), so that the composition of their prey may slightly differ from the composition of the diet of females, largely composed of benthic fishes, heavily infected by helminth larval stages (Ishikawa and Watanuki 2002). Moreover, males prey on larger fishes than do females (Kato et al. 1996). These differences should, however, also be apparent in the richness, structure, and diversity of helminth assemblages between males and females at the level of component communities; no such differences have been found. These differences are most likely present at the infracommunity level and should be much more apparent in particular species of helminths.

The taxonomic structure of the cormorant helminth fauna (dominance of digeneans relative to nematodes, tapeworms, and acanthocephalans) is characteristic of fish-eating birds; in avian hosts, a high proportion of invertebrates in the diet results in an increase in the qualitative richness of tapeworm fauna (Stock and Holmes 1987; Bush 1990; Vasileva and Georgiev 1999; Storer 2000). In the environmental conditions of northeastern Poland, the diet of great cormorant is based exclusively on fish (Stempniewicz et al. 2003a, b); in the case of the majority of detected parasites, birds become infected by eating fish which contain invasive stages (for details see, e.g., Baruš et al. 1978; Ryzhikov et al. 1985; Sitko et al. 2006; Sitko and Okulewicz 2010). Amphipods are the only source of invasive forms of the acanthocephalans *P. minutus* (Schmidt 1985). Life cycles of some species (*P. exaeretus*, *P. phalacrocoracis*, *C. aharoni*, C. *microspiculum*, *B. carbonis*, *B. rudolphii*, and *A. phalacrocoracis*) are not completely known, but some (*P. exaeretus*, *P. phalacrocoracis*, *C. aharoni*, *C. microspiculum*, and *A. phalacrocoracis*) most probably use fishes as hosts for their invasive forms (Pearson and Prévot 1985; Iskova 1985; Kanarek 2009; Sitko 2011). Life cycles of two detected species of capillarid nematodes (*B. carbonis* and *B. rudolphii*) are unknown; however, other nematodes of the genus *Baruscapillaria* detected in birds have direct life cycles with no intermediate or paratenic hosts (Moravec et al. 1987). It therefore seems unlikely that the infective stages of these nematodes may be present in fish. From this point of view, it is interesting that sexually mature individuals of a capillarid avian nematode, *Ornithocapillaria appendiculata*, typical parasite of the cormorant *Ph. brasilianus* (Moravec et al. 2000), have been detected in freshwater fish from Mexico.

Only 15 out of the 31 helminth species detected in the great cormorant can be classified as cormorant specialists. All of them, along with one captured specialist (*P. cuticola*) and two generalists (*M. xanthosomus* and *L. intestinalis*), occur in the analyzed hosts irrespective of habitat. The species identified as generalists and captured specialists occur almost exclusively in birds from the brackish water habitat which directly affects the much higher values of species richness and diversity indices observed in hosts from the Vistula Lagoon and the Vistula Spit. Distinct differences in the composition and structure of helminth assemblages between conspecific avian hosts, which occur in highly diverse habitats, are usually a consequence of the presence or availability of specific intermediate hosts and are closely associated with specific habitat conditions (Sitko 1993; Šimková et al. 2003). In the case of fish-eating birds, these differences are due to the occurrence of invertebrate intermediate or paratenic hosts (snails, arthropods, etc.) rather than to differences in prey composition between sites. Furthermore, larval stages of helminths, which mature in piscivorous birds, are generally characterized by very broad spectra of intermediate/paratenic fish hosts. The Vistula Lagoon, the main feeding area for cormorants from the breeding colony on the Vistula Spit (Bzoma et al. 2003), is typically estuarine and brackish, with distinct marine influences (Chubarenko and Margoński 2008). In contrast, lakes Wulpińske and Selment Wielki are typical inland freshwater lakes. Interestingly, despite the much higher species richness observed in cormorants from the Vistula Lagoon and the breeding colony on the Vistula Spit, only few of the recorded species can be regarded as typically associated with marine environment. Only two generalists (*C. concave* and *S. pseudoechinata*) and two captured specialists (*A. simplex* and *C. semerme*) can be regarded as characteristic marine species. The life cycles of *S. pseudoechinata* and *C. concava* are dependent on hydrobiid snails of the genera *Peringia* and *Ecrobia* (formerly *Hydrobia*) (Zander et al. 1984; Køie 1986). The nematode *A. simplex* uses planktonic Euphasiacea as the most important intermediate hosts (e.g., Klimpel et al. 2004; Levsen and Lunestad 2010), and the acanthocephalans *C. semerme* utilizes benthic arthropods of the genus *Monoporeia*, mainly *M. affinis* (e.g., Sinsalo and Valtonen 2003; Valtonen et al. 2004). Both *Hydrobia* sp. and *Monoporeia* sp. occur in the Gulf of Gdańsk and in the Vistula Lagoon (Wenne and Wiktor 1982; Herra and Wiktor 1985; Ezhova et al. 2005); Euphausiacea have not been found because of the low salinity (Grabda 1974). The occurrence of L3 *A. simplex* in adult cormorants sampled in the Vistula Lagoon in early spring results from the lagoon serving as an important spawning area for the herring *Clupea harrengus* from the West Baltic, Danish Straits, and the North Sea; the herring is heavily infected by larvae of *A. simplex* (Shukhgalter 2002) and provides a convenient source of food for the cormorant in early spring (Stempniewicz and Grochowski 1997). Interestingly, other digenean species found only in cormorants from the brackish water habitat (*A. gracilis*, *E. coaxatus*, and *E. spinulosus*) have life cycles that are associated with typically freshwater snails of the genera *Lymnea* and *Bithynia* (see Sitko et al. 2006 for review). Two species of tapeworms, classified as generalists (*D. ditremum* and *S. solidus*), are also regarded as characteristic of freshwaters (e.g., Andersen and Gibson 1989; Zander 1998; Tolonen et al. 2000), with freshwater copepods serving as intermediate hosts (see, e.g., Ryzhikov et al. 1985 for review). This is a consequence of the estuarine character of the Gulf of Gdańsk and the Vistula Lagoon, where typically freshwater and marine species co-occur. Moreover, the Gulf of Gdańsk (with Vistula Estuary) and the Vistula Lagoon are very important areas for birds during breeding and migration (Kośmicki et al. 2010; Mokwa et al. 2010). The widespread occurrence of many species of fish-eating birds may lead to accumulation of invasive forms of helminths in intermediate or paratenic hosts (Ondračková et al. 2004; Hechinger and Lafferty 2005; Fredensborg et al. 2006), which significantly increases the probability of finding helminth species that are not host specific or have a wide host spectrum (Edwards and Bush 1989). For example, the occurrence of the nematode *C. obvelatus* exclusively in cormorants from the brackish water habitat seems to result from the rather widespread occurrence of gulls and terns which are its typical final hosts, rather than from the local habitat conditions or the presence of intermediate hosts (amphipods and fishes—Wong and Anderson 1982).

Apart from the richness and diversity differences between the two habitats, they differ fundamentally in the structure of their helminth fauna. The dominance of tapeworms in birds from the freshwater habitat is due to the very high abundance of the small *P. scolecina*, which is typical of the great cormorant. The first intermediate host of *P. scolecina* is the freshwater copepod *Eudiaptomus graciloides* (Jarecka 1970). The invasive form occurs only in fishes, mainly cyprinids (Kozicka 1971). *E. graciloides* is occasionally recorded in the Gulf of Gdańsk and the Vistula Lagoon in areas adjacent to river mouths (Wiktor et al. 1982; Adamkiewicz-Chojnacka 1983; Wiktor and Żmijewska 1985); however, larvae of *P. scolecina* are only rarely found in fish from the Vistula Lagoon (Rolbiecki 2003), which suggests an overlapping limited life cycle in brackish-water habitats. The observed habitat-dependent differences in the structure of helminth assemblages may also be largely due to salinity or hydrological characteristics of the water bodies. The two freshwater lakes (Selment and Wulpinskie) are relatively large and deep, with a limited littoral zone which is very likely to facilitate transmission of helminths with life cycles involving pelagic copepods. In this context, the dominance of digeneans in the helminth fauna of birds from the brackish water habitat may be a consequence of the character of the Vistula Lagoon. The lagoon is relatively shallow, with the average depth less than 2.7 m (Chubarenko and Margoński 2008) which, combined with the rich benthic fauna (Ezhova et al. 2005), facilitates transmission of developmental stages from snails to fishes. Marine environments hold generally far richer benthic than pelagic systems (e.g., Gray 1997) which promotes a greater diversity of parasitic helminths in marine ecosystems (Marcogliese 2007).

At the component community level, the helminth assemblage of the great cormorant from northeastern Poland shows some age- and season-related variation in its richness and diversity, but in avian hosts (especially regular migrants), age and season are not independent factors. Usually, helminth assemblages of adults tend to be richer in species than those of juveniles which results from accumulation of helminth species over time and from occurrence of some helminths acquired by adult hosts in the wintering grounds (Bykhovskaya-Pavlovskaya 1962; Smogorzhevskaya 1976; Kennedy and Bakke 1989). The absence of distinct differences in species richness between adults, immature individuals, and chicks from the brackish water habitat is surprising. This unusual situation results from the large number of species of captured generalists and specialists recorded in chicks from the Vistula Spit. The differences are much more pronounced in birds from the freshwater habitat: the species richness in chicks is much smaller than in adults and immature birds, which results from the absence of several species of cormorant specialists which are exclusive to adult and immature hosts. Overall, the chick helminth assemblage shows a low specificity: there is a small proportion of cormorant specialists coupled with a large proportion of captured specialists and generalists, which may result from a handicapped immune system function in young birds (Haussmann et al. 2005; Lavoie et al. 2007; Noreen et al. 2011). It is difficult to explain the observed pattern on the basis of possible diet differences between birds of different ages: the cormorant is a typical piscivorous opportunist and exploits fish species which are locally the most abundant and the most available. Consequently, differences in the qualitative and quantitative food composition between individuals of different ages (especially between chicks and their parents) are very unlikely. The lack of well-developed immune mechanisms in young cormorants facilitates host colonization by species which are specific to other fish-eating birds and may explain the very high parameters of helminth occurrence in the oldest chicks and juveniles.

The helminth fauna of adult and immature birds includes some species which are absent in chicks (*M. yokogawai*, *C. aharonii*, *A. simplex*, *E. excisus*, *B. carbonis*, *B. rudolphii*, *A. phalacrocoracis*, *S. hispida*, and *P. minutus*); they are classified mainly as cormorant specialists (*E. excisus*, *B. carbonis*, *B. rudolphii*, and *A. phalacrocoracis*). Of these, only two species (*B. carbonis* and *B. rudolphii*) occur in adult and immature hosts independent of the season. The other species are found only in spring (*A. simplex*, *M. yokogawai*, *E. excisus*, *A. phalacrocoracis*, and *S. hispida*) or in summer (*C. aharonii* and *P. minutus*). Except for *A. simplex*, whose occurrence in spring is closely associated with the local marine influences in the Vistula Lagoon ecosystem, their presence only in spring clearly indicates their being transported by migrating cormorants from the shores of the Mediterranean. Cormorants nesting in Poland migrate regularly; their main wintering grounds are located in western and southern Europe, especially in the Mediterranean, and in North Africa (Bzoma et al. 2005). The surprising record of *C. aharonii* in immature cormorants in late summer seems not to be a consequence of the occurrence of their larval stages in northeastern Poland, but suggests a possible long survival (up to several months) of this species in its avian hosts since their return from the wintering grounds. This is confirmed by observations of Sitko (1993), who recorded *C. aharonii* in *Sterna hirundo* and *Chroicocephalus ridibundus* a few months after the end of migration. Independent of the season, the occurrence of nematodes and *B. carbonis* and *B. rudolphii* only in adult and immature hosts may indicate an effect of behavioral factors on the likelihood of infection. The life cycle of these capillarid nematodes is most probably direct (Moravec et al. 1987), and contact with soil is a prerequisite for young birds to get infected. In Central European conditions (unlike northern Europe or the British Isles), the great cormorant nests in trees, and direct transmission of these geohelminths to the chicks is difficult. After leaving the nests, the cormorants have a greater chance of contact with soil (e.g., when resting on islands, cliffs etc.), which may explain the appearance of these nematodes only in immature and adult hosts.

Having returned from their wintering grounds in early spring, the adult hosts from both habitats hold helminth assemblages which are very similar in their richness and diversity. Immediately after returning, adult cormorants start nesting and raise their chicks, which for a few months are confined to a specific habitat, so that the richness, diversity, and structure of the helminth fauna reflect the habitat quality. In the case of immature cormorants, the mechanism is much more complicated because of their nomadic behavior. The value of Brillouin’s Index of Diversity for immature cormorants, almost two times higher in spring than in summer, is especially surprising. The phenomenon can be explained by the fact that the immature cormorants sampled in spring are 2-year-old birds and were analyzed after their return from the wintering grounds (rich and diverse helminth fauna), whereas the immature birds sampled in summer were a mixture of 2-year-old and younger birds (soon after leaving their nests, in their first year of life), characterized by a much poorer and less diverse helminth fauna.

The species richness, diversity, and structure of helminth assemblages of the great cormorant at the level of component community vary widely depending on the habitat, season, and host’s age; these factors are interrelated. The interactions between the host’s sex and the richness, diversity, and structure of the helminth assemblage are much less significant. Such interactions should be much more apparent at the infracommunity level.

## Electronic supplementary material

Below is the link to the electronic supplementary material.

ESM 1 (DOCX 27 kb)
