# Supplementary material for: Extrinsic- and intrinsic-dependent variation in component communities and patterns of aggregations in helminth parasites of great cormorant (Phalacrocorax carbo) from N.E. Poland
Source: Parasitol Res. 2013 Dec 3;113(3):837–50. doi: 10.1007/s00436-013-3714-7 (PMC3932169; doi:10.1007/s00436-013-3714-7)
Supplement: Supplementary file 1 — (DOCX 27 kb) [file 436_2013_3714_MOESM1_ESM.docx]

Table listing presence, absence and status of detected helminth species in relation to habitat, season (only adult and immature cormorants) and host age. Status as coded as follows: Ps – cormorant specialist; Cs – captured specialist; G – generalist.

| Species | Status | Season | Brackish water | | |  | Freshwater | | |
| --- | --- | --- | --- | --- | --- | --- | --- | --- | --- |
|  |  |  | Adult | Immature | Chicks |  | Adult | Immature | Chicks |
| DIGENEA |  |  |  |  |  |  |  |  |  |
| *Hysteromorpha triloba* (Rudolphi, 1819) | Ps | Spring | **+** | **+** | **+** |  | **+** | **+** | **+** |
|  |  | Summer | **+** | **+** |  |  | **+** | **+** |  |
| *Posthodiplostomum cuticola* (Nordmann, 1832) | Cs | Spring | **+** | **-** | **-** |  | **+** | **+** | **-** |
|  |  | Summer | **-** | **-** |  |  | **+** | **+** |  |
| *Apatemon gracilis* (Rudolphi, 1819) | Cs | Spring | **-** | **-** | **+** |  | **-** | **-** | **-** |
|  |  | Summer | **-** | **-** |  |  | **-** | **-** |  |
| *Holostephanus dubinini* Vojtek et Vojtkova, 1968 | Ps | Spring | **+** | **+** | **+** |  | **+** | **+** | **+** |
|  |  | Summer | **+** | **+** |  |  | **+** | **+** |  |
| *Echinochasmus coaxatus* Dietz, 1909 | Cs | Spring | **-** | **-** | **+** |  | **-** | **-** | **-** |
|  |  | Summer | **-** | **-** |  |  | **-** | **-** |  |
| *Echinochasmus spinulosus* (Rudolphi, 1809) | Cs | Spring | **-** | **-** | **+** |  | **-** | **-** | **-** |
|  |  | Summer | **-** | **-** |  |  | **-** | **-** |  |
| *Stephanoprora pseudoechinata* (Olsson, 1876) | G | Spring | **-** | **+** | **+** |  | **-** | **-** | **-** |
|  |  | Summer | **-** | **-** |  |  | **-** | **-** |  |
| *Paryphostomum radiatum* (Dujardin, 1845) | Ps | Spring | **+** | **+** | **+** |  | **+** | **+** | **+** |
|  |  | Summer | **+** | **+** |  |  | **+** | **+** |  |
| *Petasiger exaeretus* Dietz, 1909 | Ps | Spring | **+** | **+** | **+** |  | **+** | **+** | **+** |
|  |  | Summer | **+** | **+** |  |  | **+** | **+** |  |
| *Petasiger phalacrocoracis* (Yamaguti, 1939) | Ps | Spring | **+** | **+** | **+** |  | **+** | **+** | **+** |
|  |  | Summer | **+** | **+** |  |  | **+** | **+** |  |
| *Cercarioides aharonii* Witenberg, 1929 | G | Spring | **-** | **-** | **-** |  | **-** | **-** | **-** |
|  |  | Summer | **-** | **+** |  |  | **-** | **-** |  |
| *Cryptocotyle concava* (Creplin, 1825) | G | Spring | **+** | **+** | **+** |  | **-** | **-** | **-** |
|  |  | Summer | **+** | **-** |  |  | **-** | **-** |  |
| *Metagonimus yokogawai* Katsurada, 1913 | G | Spring | **-** | **+** | **-** |  | **-** | **-** | **-** |
|  |  | Summer | **-** | **-** |  |  | **-** | **-** |  |
| *Metorchis xanthosomus* (Creplin, 1846) | G | Spring | **+** | **+** | **+** |  | **+** | **+** | **+** |
|  |  | Summer | **+** | **+** |  |  | **+** | **+** |  |
| CESTODA |  |  |  |  |  |  |  |  |  |
| *Diphyllobothrium ditremum* (Creplin, 1846) | G | Spring | **+** | **+** | **+** |  | **-** | **-** | **-** |
|  |  | Summer | **+** | **-** |  |  | **-** | **-** |  |
| *Ligula intestinalis* (L., 1758) | G | Spring | **+** | **-** | **+** |  | **+** | **-** | **+** |
|  |  | Summer | **-** | **-** |  |  | **-** | **+** |  |
| *Schistocephalus solidus* (Müller, 1776) | G | Spring | **+** | **+** | **+** |  | **-** | **-** | **-** |
|  |  | Summer | **+** | **-** |  |  | **-** | **-** |  |
| *Paradilepis scolecina* (Rudolphi, 1819) | Ps | Spring | **+** | **+** | **+** |  | **+** | **+** | **+** |
|  |  | Summer | **+** | **+** |  |  | **+** | **+** |  |
| NEMATODA |  |  |  |  |  |  |  |  |  |
| *Cyathostoma microspiculum* (Skrjabin, 1915) | Ps | Spring | **+** | **+** | **+** |  | **+** | **-** | **-** |
|  |  | Summer | **+** | **+** |  |  | **-** | **+** |  |
| *Anisakis simplex* L3 (Rudolphi, 1809) | Cs | Spring | **+** | **-** | **-** |  | **-** | **-** | **-** |
|  |  | Summer | **-** | **-** |  |  | **-** | **-** |  |
| *Contracaecum rudolphii* Hartwich, 1964 | Ps | Spring | **+** | **+** | **+** |  | **+** | **+** | **+** |
|  |  | Summer | **+** | **+** |  |  | **+** | **+** |  |
| *Cosmocephalus obvelatus* (Creplin, 1825) | Cs | Spring | **-** | **-** | **+** |  | **-** | **-** | **-** |
|  |  | Summer | **-** | **-** |  |  | **-** | **-** |  |
| *Syncuaria squamata* (Linstow, 1883) | Ps | Spring | **+** | **+** | **+** |  | **+** | **-** | **+** |
|  |  | Summer | **+** | **+** |  |  | **+** | **+** |  |
| *Desmidocercella incognita* Solonitsin, 1932 | Ps | Spring | **+** | **+** | **+** |  | **+** | **+** | **+** |
|  |  | Summer | **+** | **+** |  |  | **+** | **+** |  |
| *Eustrongylides excisus* Jägerskiöld, 1909 | Ps | Spring | **+** | **+** | **-** |  | **+** | **-** | **-** |
|  |  | Summer | **-** | **-** |  |  | **-** | **-** |  |
| *Baruscapillaria carbonis* (Dubinin et Dubinina, 1940) | Ps | Spring | **+** | **+** | **-** |  | **+** | **+** | **-** |
|  |  | Summer | **+** | **+** |  |  | **+** | **+** |  |
| *Baruscapillaria rudolphii* Moravec, Scholz et Našincová, 1994 | Ps | Spring | **+** | **+** | **-** |  | **+** | **+** | **-** |
|  |  | Summer | **+** | **+** |  |  | **+** | **+** |  |
| ACANTHOCEPHALA |  |  |  |  |  |  |  |  |  |
| *Polymorphus minutus* Skrjabin, 1913 | Cs | Spring | **-** | **-** | **-** |  | **-** | **-** | **-** |
|  |  | Summer | **-** | **-** |  |  | **-** | **+** |  |
| *Corynosoma semerme* (Forssell, 1904) | Cs | Spring | **-** | **-** | **+** |  | **-** | **-** | **-** |
|  |  | Summer | **-** | **-** |  |  | **-** | **-** |  |
| *Andracantha phalacrocoracis* (Yamaguti, 1939) | Ps | Spring | **+** | **-** | **-** |  | **+** | **+** | **-** |
|  |  | Summer | **-** | **-** |  |  | **-** | **-** |  |
| *Southwellina hispida* (van Cleave, 1925) | G | Spring | **+** | **+** | **-** |  | **-** | **-** | **-** |
|  |  | Summer | **-** | **-** |  |  | **-** | **-** |  |
